# Oatmeal induced gut microbiota alteration and its relationship with improved lipid profiles: a secondary analysis of a randomized clinical trial

**DOI:** 10.1186/s12986-020-00505-4

**Published:** 2020-10-08

**Authors:** Mengyao Ye, Jianqin Sun, Yanqiu Chen, Qian Ren, Zhen Li, Yanfang Zhao, Yiru Pan, Huijun Xue

**Affiliations:** 1 Clinical Nutrition Center, Huadong Hospital Affiliated to Fudan University, 221 West Yan’an Road, Shanghai, 200040 People’s Republic of China; 2 Huadong Hospital Affiliated to Fudan University, 221 West Yan’an Road, Shanghai, 200040 People’s Republic of China

**Keywords:** Oatmeal, Hypercholesterolemia, Microbiota, 16SrRNA

## Abstract

**Background:**

In vitro and animal experiments reported a microbiota-regulating ability of oatmeal, however, related in vivo evidences remained limited. Thus, we conducted this study aiming to investigate the oatmeal-induced alteration of gut microbiota and its potential relationship with the improvements of lipid profiles.

**Methods and study design:**

Data of anthropometric measurements and biochemical parameters were extracted from a randomized, controlled clinical trial, in which 62 hypercholesterolemic men and women (18–65 years old) were provided with either treatment of 80 g/day oatmeal or 80 g/day refined white rice for 45 days. Fasting blood samples and fecal samples were collected both at baseline and endpoint of the study for lipid profiling and microbiota 16S rRNA amplicon sequencing, respectively.

**Results:**

Totally 28 participants (56 fecal samples) qualified with the new criteria and were thus included in this secondary analysis. The results of microbiota analysis showed that no significant difference was observed in the alteration of its overall α or β diversity between two groups throughout the study. Nor did any notable between-group difference was found in the relative abundance changes of microorganism at different taxonomies. However, results from linear discriminant analysis effect size in the oatmeal group indicated a significant positive response of Firmicutes phylum following oatmeal consumption. Further Procrustes analysis suggested a concordance trend between microorganism alteration and alleviation of hypercholesterolemia phenotypes throughout the study (*P* = 0.05). The results of within-group comparison from Spearman’s correlation in the oatmeal group demonstrated a significant association between the enrichment of Blautia genus and the reduction of serum total cholesterol (*P* < 0.05), low-density lipoprotein cholesterol (*P* < 0.01), and apolipoprotein B (*P* < 0.05).

**Conclusions:**

Positive response of Firmicutes phylum might be a critical characteristic of oatmeal-induced alteration of microbiota, whereas, one of the underlying cholesterol-lowering mechanism of oatmeal consumption might be its microbiota-manipulating ability, in which the enrichment of Blautia genus played a potentially significant role. Current results should be taken cautiously and more studies were needed for further verification.

**Trial registration**: ChiCTR, ChiCTR180001864. Registered 30 September 2018, http://www.chictr.org.cn/showproj.aspx?proj=31469.

## Introduction

Oatmeal, being abundant with fermentable fiber, was assumed to be able to influence the composition of microbiota by many ways, such as utilizing the discrepancy of processing capacity in different energy substrates or changing intestinal microenvironment like pH [1]. Meanwhile, current evidences supported that gut microbiota regulation toward enhancing proportion of beneficial members of microorganism community might be a novel and promising ecological strategy for hypercholesterolemia management [2–4]. These evidences provided a new perspective, that oatmeal was potentially able to regulate microbiota effectively and its microbiota-manipulating ability might be an underlying mechanism of its hypocholesterolaemic effect.

Most of animal experiments have suggested a prebiotic effect of oatmeal consumption [5, 6]. However, in vitro fermentation researches tended to show contradictory results, with some being consistent with the previous animal experiments [7, 8], yet others the opposite [9, 10]. Limited clinical trials also reported mixed results due to inconsistent microbiota testing methodologies, experimental models and study designs [11–13]. Moreover, whether the oatmeal-induced microbiota alteration was related to the cholesterol benefits of oatmeal consumption also remained unexplored.

Consequently, based on a randomized, parallel, controlled trial, we further tested the fecal gut microbiota and did this secondary analysis, aiming to investigate the oatmeal-induced gut microbiota alterations and further explore its relationship with improvements of lipid profiles.

## Materials and methods

### Participants and study design

The data of clinical parameters and the fecal samples both came from the randomized, controlled, parallel trial conducted at Huadong hospital in Shanghai, which mainly focused on the cholesterol-lowering effect of oatmeal consumption. The study was conducted in accordance with the Declaration of Helsinki and all participants were given informed consent before participation. The study protocol was registered in the Chinese Clinical Trial Registry (Identifier: ChiCTR180001864) and was approved by the Institutional Review Board of Huadong Hospital (No. 20180059).

The detailed study design of the clinical trial has been described before [14]. Briefly, 62 men and women aged 18–65 years old with mild to moderate hypercholesterolemia (5.2 mmol/L ≤ total cholesterol [TC] ≤ 6.8 mmol/L; triglyceride [TG] < 2.3 mmol/L) were recruited. Principal exclusion criteria including: having hypocholesterolaemic medication; having diabetes; having heart, liver, kidney, gastrointestinal, or hematopoietic system diseases, or being mentally ill; with a body mass index (BMI) ≥ 28 kg/m^2^; having had a regular intake (≥ 3 times per week) of oatmeal or other foods rich in β-glucan in the past 6 months; any dietary restrictions that would affect trial completion. And to ensure the effectiveness of the microbiota analysis of this study, two extra inclusion criteria were added as follows: (1) without a history of antibiotics intake within 3 months before the beginning of the study as well as throughout the study; (2) providing qualified fecal samples both at baseline and endpoint of the study.

### Interventions and measurements

Participants were equally randomized to consume either 80 g/d oatmeal (oatmeal group) or an equal amount of refined white rice (control group) substituting part of their staple food, whereas under a context of keeping in a habitual dietary pattern and physical activity for 45 days. The compositions of two food were presented in Table 1. Considering the common food intake amount of Chinese and the fact that most of participants were office workers who usually ate their lunch at canteens or restaurants, participants were required to consume the given food at breakfast and dinner (40 g per meal) to ease the difficulty of realistic execution. The participants were instructed to boil the oatmeal or refined white rice with hot water before consumption. All the above cooking and consumption directions were given by well-trained researchers before the beginning of the study. To record the corresponding food consumption, adverse events and drug usage during the study, telephone follow-ups were carried out weekly, whereas face-to-face visit was carried out every 2 weeks. The oatmeal and refined white rice were provided in an amount that was sufficient for 2 weeks each time (at the last visit, the provided amount was expected to be sufficient for 17 days), so the food was also replenished during the face-to-face visit.

**Table 1:** Composition of oatmeal and refined white rice (per 80 g)

Nutrients	Oatmeal	Control
Energy (Kcal)	304	311
Protein (g)	8.8	5.3
Fat (g)	7.4	1.5
Carbohydrate (g)	48.4	67.7
Total fiber (g)	9.6	0.7
β-glucan (g)	3.0	0.0

Fasting venous blood samples and fecal samples were collected both at baseline and the endpoint. Serum was further extracted and stored at − 80 °C until further lipid profile testing, which involved the assessments of TC, TG, low-density lipoprotein cholesterol (LDL-C), high-density lipoprotein cholesterol (HDL-C), small dense low-density lipoprotein cholesterol (sdLDL-C) and apolipoprotein B (apoB). All lipid profiles were tested by using commercial kits with the help of fully automated analyzer. Non-high-density lipoprotein cholesterol (non-HDL-C), which mainly consisted of LDL-C and very-low-density lipoprotein cholesterol, were calculated as the absolute concentration difference between TC and HDL-C. Collected fecal samples were also stored at − 80 °C until further deoxyribonucleic acid (DNA) extracting and sequencing. Dietary intakes and physical activity were monitored both at baseline and the endpoint as potential confounding factors by conducting 3 days 24-h dietary recall and physical activity questionnaires, respectively. And dietary intakes were calculated on the basis of the nutrients content in the *China food composition (2002)* [15], but the composition data of oatmeal and refined white rice at endpoint were using the corresponding data in this study.

**Fig. 1:**
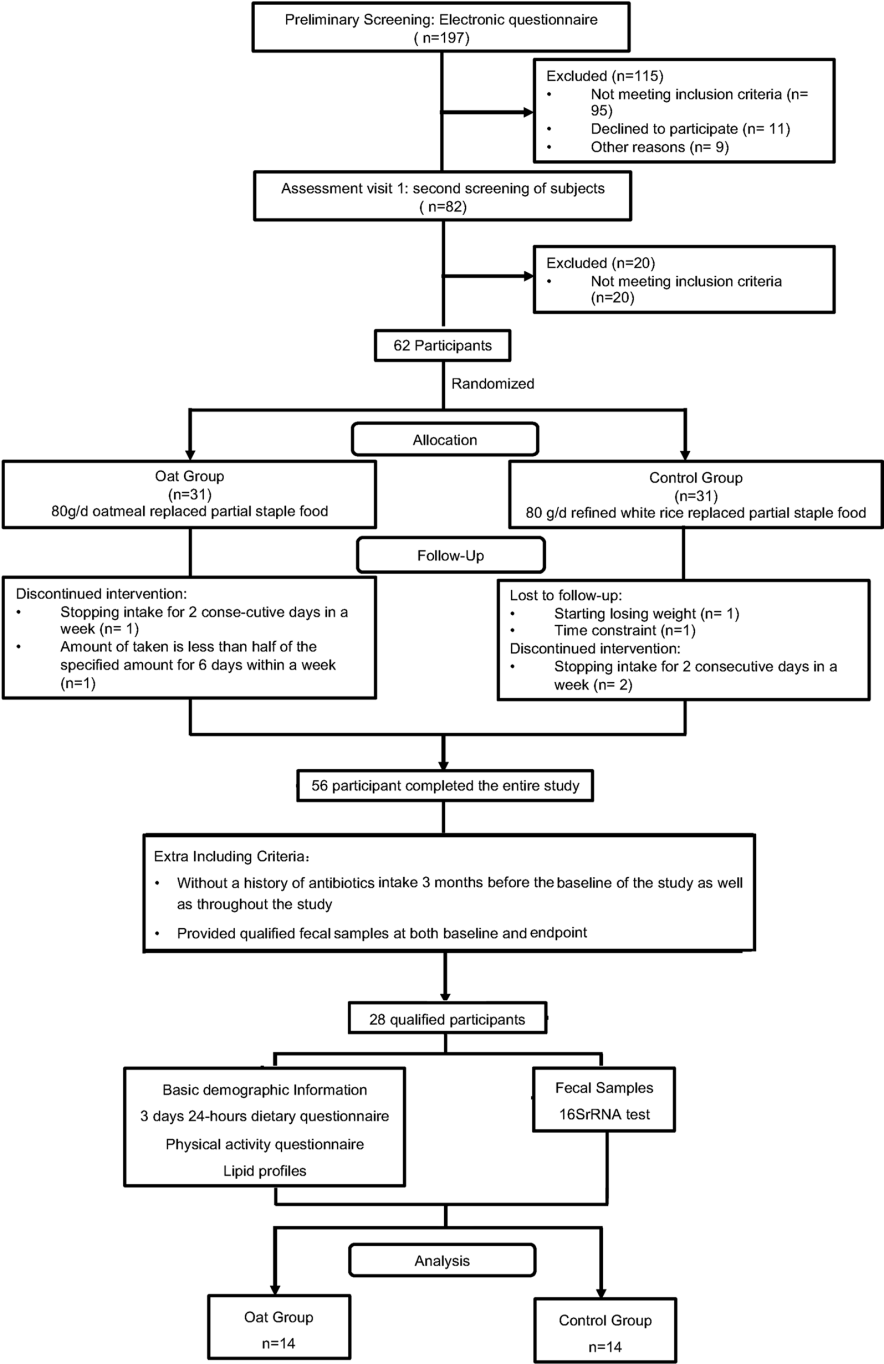
Flow chart

Finally, 28 (14 per group) out of 62 participants were qualified and included in this secondary analysis. The flow chart was shown in the Fig. 1.

### DNA extraction, polymerase chain reaction (PCR) amplification and Miseq sequencing

Totally 56 samples were included for 16S rRNA sequencing. The PCR amplification and Illumina sequencing process was similar to a previous study [16]. In brief, microbial DNA was extracted from stool samples using QIAamp DNA Stool Mini Kit protocol (Qiagen, Germany). The V3–V4 regions of the 16S rRNA gene were amplified by using the primer pair of 357F 5′-ACTCCTACGGRAGGCAGCAG-3′ and 806R 5′-GGACTACHVGGGTWTCTAAT-3′. Another two pairs of primers contained the Illumina 5′ overhang adapter sequences for two-step amplicon library building, and all processes followed the instructions of manufacturer for the overhang sequences. The amplified DNA products were verified by agarose gel electrophoresis and recovered by using AxyPrepDNA Gel Recovery Kit (Axygen, China). They were then quantified using FTC-3000TM real-time PCR and sequenced on the MiSeq platform by 2 * 300 bp paired-end sequencing using MiSeq v3 Reagent Kit (Illumina, USA). The raw reads were deposited into the NCBI Sequence Read Archive database (Accession number: SRP230372).

### Library construction

The raw fastq files were demultiplexed based on the barcode. Trimmomatic (0.35) [17] was used for quality filtering (low-quality base pairs removing parameters: Slidingwindow: 50:20 and Minlen: 50) and FLASH program (1.2.11) [18] was used for reads merge (parameters: minimum overlap length = 10 bp, maximum ratio of mismatch = 0.2). Low-quality contigs were removed by using Mothur (1.39.5) (filtering parameters: maxambig = 0, minlength = 200, maxlength = 580, maxhomop = 8). UPARSE pipeline (https://drive5.com/usearch/manual/uparse cmds.html) was then used for operational taxonomic unit (OTU) clustering with a pairwise identity threshold of 97% and UCHIME was used for chimeric reads filtering [19]. Taxonomically classification of representative OTU sequences was assignment by using Mothur (1.39.5) in according to Silva 128 database (confidence score threshold = 0.6). Unannounced OTUs were removed prior to statistical analysis. The phylogenetic tree was built by using FastTree (2.1.3), [20] and the acquired OTU table as well as the phylogenetic tree were used for diversity analysis.

### Fecal microbiota analysis

The statistician kept blinded to the treatments. The richness and diversity of microbiota composition were evaluated by the Ace index and Shannon index of α diversity by using R (3.6.0). Bray–Curtis dissimilarity distance was calculated by Mothur (1.39.5). Principal coordinate analysis (PCoA) was based on the Bray–Curtis dissimilarity distance of 448 OTUs. Unweighted pair-group method with arithmetic mean (UPGMA) based on Bray–Curtis dissimilarity distance of 448 OTUs was used for clustering analysis. Procrustes analysis was also performed in R (3.6.0) by using the vegan package, and Monte Carlo *P* values for rotational agreement significance testing was determined from 999 permutations. Between-group differences in the relative abundance alterations of microorganism (defined as the difference from baseline) at different taxonomies during study period were evaluated by using multiple test corrected by Benjamini–Hochberg false discovery rate (FDR), with Q value of 0.20 being set as significance. Whereas, the data of microorganism alterations used in the Spearman correlation analysis was calculated from log-transformed fold changes between microbial relative abundance at 45 day against those at 0 day. And before deriving fold changes, the zero values were first additively smoothed by the minimal non-zero abundance among all observed measurements within the group. The data of lipid profile changes used in Spearman correlation analysis was also calculated as the log-transformed fold changes between baseline and endpoint concentrations. The data of microbiota was not normally distributed and unless otherwise indicated, Mann–Whitney U test (two-tailed) and Wilcoxon signed-rank tests (two-tailed) were used throughout the study for unpaired and paired sample comparisons, respectively. All statistical analysis of microbiota data was performed in R (3.6.0), unless otherwise stated. The pwr package and HMP package in R (3.6.0) were used to conduct post-hoc power calculations with a significance level being set to 0.05 and a sample size of 14 per group. The results were shown in the Additional file 1: Supplementary Table 1.

### Clinical parameters statistical analysis

The statistician kept blinded to the treatments. All parameters were expressed as mean ± standard error (SE). Unless otherwise indicated, the continuous data with normal distribution and homogenous variance was statistically tested by using independent *t* test (between-group comparisons) and paired *t* test (within-group comparisons), respectively; whereas the other continuous data was statistically tested by using Mann–Whitney U test (between-group comparisons) and Wilcoxon Signed rank test (within-group comparisons) respectively. Chi-square test was used for between-group comparison when analyzing classify variables. For data of dietary intakes and physical activity, comparisons between changes from baseline were analysed in order to minimize the bias of baseline differences. Between-groups differences of lipid changes were evaluated by using analysis of covariance (ANCOVA) after taking baseline concentration of lipid profiles and any other unbalanced baseline measurements as covariates. Besides, any significantly different changes of dietary intakes and physical activity between two groups were also taken as covariates if with necessity. SPSS (23.0) were used for statistical analysing in this part. A value of *P* < 0.05 (two-tailed) was considered significant for all statistical analysis.

## Results

### Baseline characteristics

The average eating amount of corresponding testing food were 78.1 ± 0.9 g/day and 76.4 ± 1.6 g/day in the oatmeal and the control group, respectively, which indicated a satisfactory compliance by all participants. Only two participants (both from the oatmeal group) reported various degrees of gastrointestinal reactions like bloating and frequent fart, yet all basically relieved within 3 weeks. The quite high consuming compliance and the quite low incidence of adverse events indicated a good feasibility of substituting parts of staple food with 80 g/day oatmeal toward increased dietary fiber intake in the population of current study. Between-group imbalances of TG (1.44 ± 0.13 mmol/L vs. 1.08 ± 0.07 mmol/L, *P* = 0.02), sdLDL-C (1.06 ± 0.12 mmol/L vs. 0.73 ± 0.04 mmol/L, *P* = 0.02) and every-day working time (8.86 ± 0.41 h/day vs. 6.14 ± 0.94 h/day, *P* = 0.01) were observed at baseline. Besides, other baseline characteristics kept comparable between two groups (Table 2).

**Table 2:** Baseline characteristics of participants

Variable	Oatmeal (n = 14)	Control (n = 14)	*P* value
Age	45.7 ± 2.5	49.4 ± 2.6	0.32
Gender (men/women)	7/7	6/8	1.00^a^
Occupation (medical/office worker/others)	10/2/2	6/6/2	–
Body weight (kg)	64.8 ± 2.2	64.4 ± 2.9	0.93
Waistline (cm)	83.3 ± 1.6	82.3 ± 1.7	0.68
Hip circumference (cm)	96.3 ± 1.4	94.8 ± 1.3	0.46
Systolic blood pressure (mmHg)	122.3 ± 3.4	119.2 ± 3.2	0.51
Diastolic blood pressure (mmHg)	72.0 ± 2.2	71.5 ± 2.9	0.57^b^
BMI (kg/m^2^)	23.5 ± 0.6	23.6 ± 0.7	0.92
WHR	0.87 ± 0.01	0.87 ± 0.02	0.85
TC (mmol/L)	6.03 ± 0.08	5.79 ± 0.09	0.06
TG (mmol/L)	1.44 ± 0.13	1.08 ± 0.07	0.02
LDL-C (mmol/L)	3.64 ± 0.12	3.40 ± 0.13	0.17
HDL-C (mmol/L)	1.68 ± 0.07	1.69 ± 0.07	0.92
non-HDL-C (mmol/L)	4.35 ± 0.11	4.11 ± 0.10	0.11
sdLDL-C (mmol/L)	1.06 ± 0.12	0.73 ± 0.04	0.02
apoB (mmol/L)	1.16 ± 0.04	1.07 ± 0.03	0.09
Energy (kcal/day)	1414.1 ± 80.1	1425.7 ± 110.4	0.84^b^
Carbohydrate (g/day)	146.5 ± 11.0	156.4 ± 16.9	0.98^b^
Fat (g/day)	64.8 ± 3.5	61.1 ± 2.9	0.48^b^
Protein (g/day)	61.2 ± 5.0	62.5 ± 5.9	0.84^b^
Fiber (g/day)	7.1 ± 1.4	5.8 ± 1.2	0.47
Cholesterol (mg/day)	259.3 ± 31.6	306.8 ± 42.8	0.38
Every-week working day (day/week)	5.18 ± 0.10	3.86 ± 0.58	0.10^b^
Every-day working time (h/day)	8.86 ± 0.41	6.14 ± 0.94	0.01^b^
Sitting time (h/day)	7.54 ± 0.73	6.46 ± 0.64	0.28
Commuting time (min/day)	74.6 ± 10.5	91.4 ± 17.4	0.70^b^
Weekly frequency of medium intensity exercise	0.17 ± 0.10	0.24 ± 0.08	0.35^b^
Weekly frequency of heavy intensity exercise	0.06 ± 0.03	0.11 ± 0.05	0.64^b^
House working time (min/day)	36.9 ± 13.6	66.8 ± 17.0	0.09^b^
Sleeping time (h/day)	6.64 ± 0.18	6.79 ± 0.25	0.80^b^

Values were presented as mean ± SE

^a^Chi-square test

^b^Mann–Whitney U test (two-tailed). Otherwise, independent-sample *t* test (two-tailed) was used

### Changes of dietary intakes and physical activity during study period

Compared to the control group, the fiber intake was notably increased in the oatmeal group throughout study (6.7 ± 1.5 g/day vs. 0.6 ± 1.4 g/day, *P* < 0.01), and results from within-group comparisons confirmed the critical role of fiber intake surge from the oatmeal group (7.12 ± 1.41 g/day vs. 13.78 ± 0.73 g/day, *P* < 0.01) in accounting for this significant between-group difference. Besides, no other significant within- or between-group difference was seen in nutrients intakes throughout the study (Table 3). And no significant difference was seen in the changes of any physical activities between two groups during study period (Additional file 1: Supplementary Table 2).

**Table 3:** Changes of dietary intakes in the oatmeal and control group throughout the study

Variables	Group	Day 0	Day 45	*P* value	Change from baseline	*P* value time by treated
Energy (kcal/day)	Oatmeal	1414.1 ± 80.2	1531.6 ± 106.0	0.20^b^	117.5 ± 83.8	0.78
	Control	1425.7 ± 110.4	1579.5 ± 136.9	0.20^b^	153.8 ± 95.2	
Carbohydrate (g/day)	Oatmeal	146.5 ± 11.0	167.6 ± 17.8	0.15	21.0 ± 13.6	0.42
	Control	156.4 ± 16.9	196.4 ± 25.1	0.07^b^	40.0 ± 18.9	
Fat (g/day)	Oatmeal	64.8 ± 3.5	68.5 ± 3.5	0.22^b^	3.67 ± 3.20	0.19
	Control	61.1 ± 2.9	60.0 ± 2.7	0.47^b^	− 1.14 ± 1.61	
Protein (g/day)	Oatmeal	61.2 ± 5.0	61.3 ± 3.4	0.64^b^	0.10 ± 5.14	0.89
	Control	62.5 ± 5.9	63.5 ± 5.5	0.55^b^	1.01 ± 3.90	
Fiber (g/day)	Oatmeal	7.12 ± 1.41	13.78 ± 0.73	< 0.01	6.65 ± 1.47	< 0.01^a^
	Control	5.75 ± 1.21	6.34 ± 1.35	0.83^b^	0.61 ± 1.35	
Cholesterol (mg/day)	Oatmeal	259.3 ± 31.6	220.3 ± 33.0	0.17	− 39.0 ± 27.0	0.64
	Control	306.8 ± 42.8	292.4 ± 33.2	0.75	− 14.4 ± 43.8	

Values were presented as mean ± SE

^a^*P* value time by treated was obtained from Mann–Whitney U test (two-tailed), otherwise, it was obtained from independent-sample *t* test (two-tailed)

^b^*P* value was obtained from Wilcoxon signed rank test (two-tailed), otherwise, it was obtained from paired *t* test (two-tailed)

### Changes of lipid profiles during study period

Our data indicated that, consistent with the results of the primary trial, significant reductions were observed in the TC (− 1.00 ± 0.12 mmol/L vs. − 0.43 ± 0.10 mmol/L, *P* < 0.01), LDL-C (− 0.29 ± 0.09 mmol/L vs. 0.20 ± 0.07 mmol/L, *P* < 0.01), non-LDL-C (− 0.97 ± 0.11 mmol/L vs. − 0.41 ± 0.10 mmol/L, *P* = 0.02), sdLDL-C (− 0.19 ± 0.08 mmol/L vs. 0.23 ± 0.05 mmol/L, *P* = 0.02) and apoB (− 0.13 ± 0.02 mmol/L vs. 0.02 ± 0.02 mmol/L, *P* = 0.01) after oatmeal consumption when compared to the control group. Whereas, no significant between-group difference was seen in the regulation of TG or HDL-C (Table 4).

**Table 4:** Changes of lipid profiles in the oatmeal and control group throughout the study

Variables (mmol/L)	Group	Day 0	Day 45	*P* value	Change from baseline	*P* value time by treated^b^
TC	Oatmeal	6.03 ± 0.08	5.03 ± 0.13	< 0.01	− 1.00 ± 0.12	< 0.01
	Control	5.79 ± 0.09	5.37 ± 0.12	< 0.01	− 0.43 ± 0.10	
TG	Oatmeal	1.44 ± 0.13	1.07 ± 0.08	< 0.01	− 0.37 ± 0.09	0.11
	Control	1.08 ± 0.07	1.31 ± 0.16	0.25^a^	0.23 ± 0.13	
LDL-C	Oatmeal	3.64 ± 0.12	3.35 ± 0.12	< 0.01	− 0.29 ± 0.09	< 0.01
	Control	3.40 ± 0.13	3.60 ± 0.13	0.01	0.20 ± 0.07	
HDL-C	Oatmeal	1.68 ± 0.07	1.65 ± 0.07	0.37	− 0.03 ± 0.03	0.14
	Control	1.69 ± 0.07	1.67 ± 0.07	0.80	− 0.02 ± 0.06	
Non-HDL-C	Oatmeal	4.35 ± 0.11	3.38 ± 0.11	< 0.01	− 0.97 ± 0.11	0.02
	Control	4.11 ± 0.10	3.69 ± 0.13	< 0.01	− 0.41 ± 0.10	
sdLDL-C	Oatmeal	1.06 ± 0.12	0.87 ± 0.08	0.04	− 0.19 ± 0.08	0.02
	Control	0.73 ± 0.04	0.96 ± 0.07	< 0.01	0.23 ± 0.05	
apoB	Oatmeal	1.16 ± 0.04	1.04 ± 0.03	< 0.01	− 0.13 ± 0.02	0.01
	Control	1.07 ± 0.03	1.10 ± 0.03	0.30^a^	0.02 ± 0.02	

Values were presented as mean ± SE

^a^*P* value was obtained from Wilcoxon signed rank test (two-tailed), otherwise, it was obtained from paired *t* test (two-tailed)

^b^*P* value time by treated was obtained from ANCOVA, where baseline concentration of lipid profiles and the level of every-day working time were taken as covariates

### Compositional alterations of bacterial community during study period

**Fig. 2:**
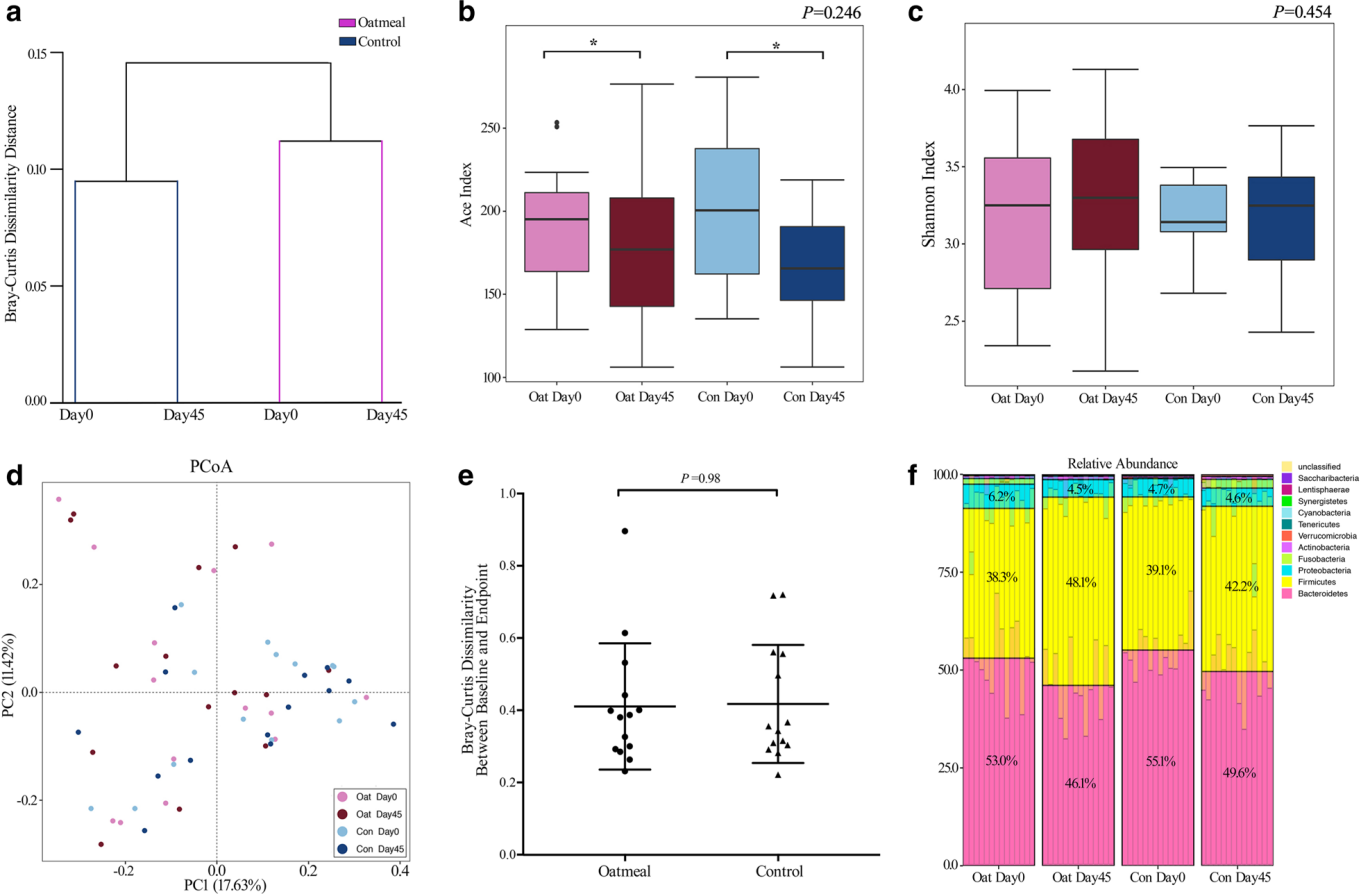
Diversity analysis of microorganism in the oatmeal and control group during study period. (**a**) Clustering analysis of gut microbiota. UPGMA was used based on Bray–Curtis dissimilarity distance of 448 OTUs. (**b**) Ace index. (**c**) Shannon index. Boxes showed the medians and the interquartile ranges (IQRs), the whiskers denoted the lowest and highest values that were within 1.5 times the IQR from the first and third quartiles, and outliers were shown as individual black dots. Wilcoxon signed-rank test (two-tailed) was used for within-group comparisons; whereas, Mann–Whitney U test (two-tailed) was used for between-group comparisons. * indicated a *P* value under 0.05. Whereas, the *P* value in the right upper corner was from Mann–Whitney U test (two-tailed) evaluating the difference of the index alteration throughout the study between two groups. (**d**) PCoA for the oatmeal and control group at baseline and endpoint based on Bray–Curtis dissimilarity distance of 448 OTUs. PC1, principal coordinate 1; PC2, principal coordinate 2. (**e**) Bray–Curtis dissimilarity distance (on the basis of 448 OTUs) between baseline and endpoint in the oatmeal and control group. Mann–Whitney U test was used for statistical analyzing. (**f**) The microbiota composition at phylum level in both groups. Oat Day 0: baseline in oatmeal group; Oat Day 45: endpoint in oatmeal group; Con Day 0: baseline in control group; Con Day 45: endpoint in control group

A total of 1,929,466 sequences were obtained with an average of 34,455** ± **4446 reads per sample. Totally 448 OTUs were obtained at a similarity level of 97%, and 11 phyla, 103 genera as well as 138 species were identified in this study. The result of clustering analysis demonstrated a more similar gut flora composition within group throughout the study, indicating that the giant heterogeneity of individuals outweighed the interventions and played a more critical role in the between-group differences of gut microbiota composition in this study (Fig. 2a). Therefore, our further analysis mainly focused on the respective microbiota alterations within group during study period, and saw if there were any differences between them.

Our data showed that no significant between-group difference was seen in the alteration of Ace index or Shannon index during study period (Fig. 2b, c), nor was any notable difference observed in the β diversity of the overall microbiota composition between groups throughout the study (Fig. 2d, e). Besides, between-group comparisons involving relative abundance alterations of microorganism at different taxonomies showed that no significant difference was observed in the alteration of microbiota composition from phylum to species levels between two groups during study period (FDR > 0.20, data not show). However, a transition of the first predominant-phylum, from Bacteroides to Firmicutes, was observed within oatmeal group throughout the study (Fig. 2f), along with a significant decrease in the relative abundance of Bacteroides phylum (7.0% ± 2.7%, *P* = 0.02) and Proteobacteria phylum (− 1.7% ± 0.8%, *P* = 0.03), while a notable increase in the relative abundance of Firmicutes phylum (9.8% ± 3.2%, *P* < 0.01) in the oatmeal group during study period. Whereas, such notable transition was not observed in the control group (Additional file 1: Supplementary Table 3).

**Fig. 3:**
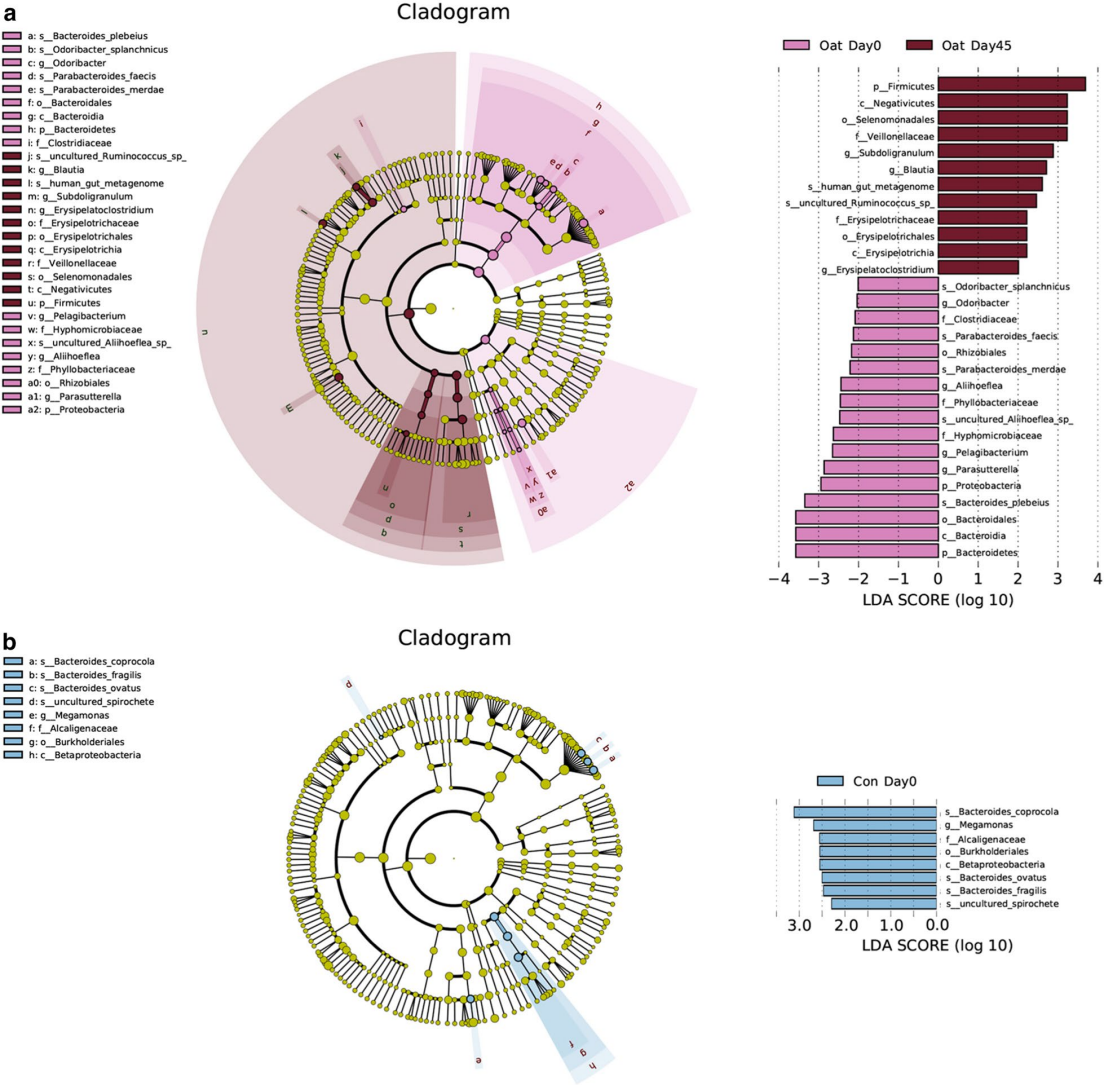
Results of LEfSe in two groups during the study period. (**a**) Oatmeal group; (**b**) Control group. Wilcoxon signed rank test was used for statistical analyzing. The threshold of LDA score was set to 2.0. The regions marked with yellow indicated no significant difference between baseline and endpoint within group. Light pink and dark pink circles and nodes designated microorganism that were significantly decreased and increased after treatment in the oatmeal group, respectively; while light blue and dark blue circles and nodes designated microorganism that were significantly decreased and increased after treatment in the control group, respectively. Oat Day 0: baseline in oatmeal group; Oat Day 45: endpoint in oatmeal group; Con Day 0: baseline in control group; Con Day 45: endpoint in control group

This result drove us to consider that the quite small sample size (n = 14 per group) and the consequently accompanied low detecting power had limited the ability to detect the independent microbiota-regulating effect of oatmeal in this study and thus, might be the reason to blame for the above negative results. Therefore, we further made within-group comparisons by using Wilcoxon signed rank test, trying to trace the significantly altered microbiomes in the oatmeal and control group, respectively, and saw if it could provide any clues for the tendencies of oatmeal induced microbiota alterations. Simultaneously, borrowed from the analyzing idea of linear discriminant analysis effect size (LEfSe), a threshold of logarithmic linear-discriminant analysis (LDA) score being set to 2.0 was also combined during screening process, ensuring to obtain the potential microorganism with real influence. The results showed that, at genus level, 3 positive responders (i.e., the relative abundance of them were significantly increased after intervention), including Blautia, Erysipelatoclostridium and Subdoligranulum (all from Firmicutes phylum), as well as 3 negative responders (i.e., the relative abundance of them were significantly decreased after intervention), including Odoribacter (from Bacteroidetes phylum) and Aliihoeflea, Pelagibacterium (both from Proteobacteria phylum), were found in the oatmeal group (Fig. 3a). Whereas, in the control group, only one negative responder (Megamonas genus from Bacteroidetes phylum) was observed at genus level (Fig. 3b). These results, to some extent, reconfirmed the critically role of Firmicutes phylum as a positive responder in the oatmeal group.

### Associations between alterations of gut microbiota and improvements of lipid profiles

**Fig. 4:**
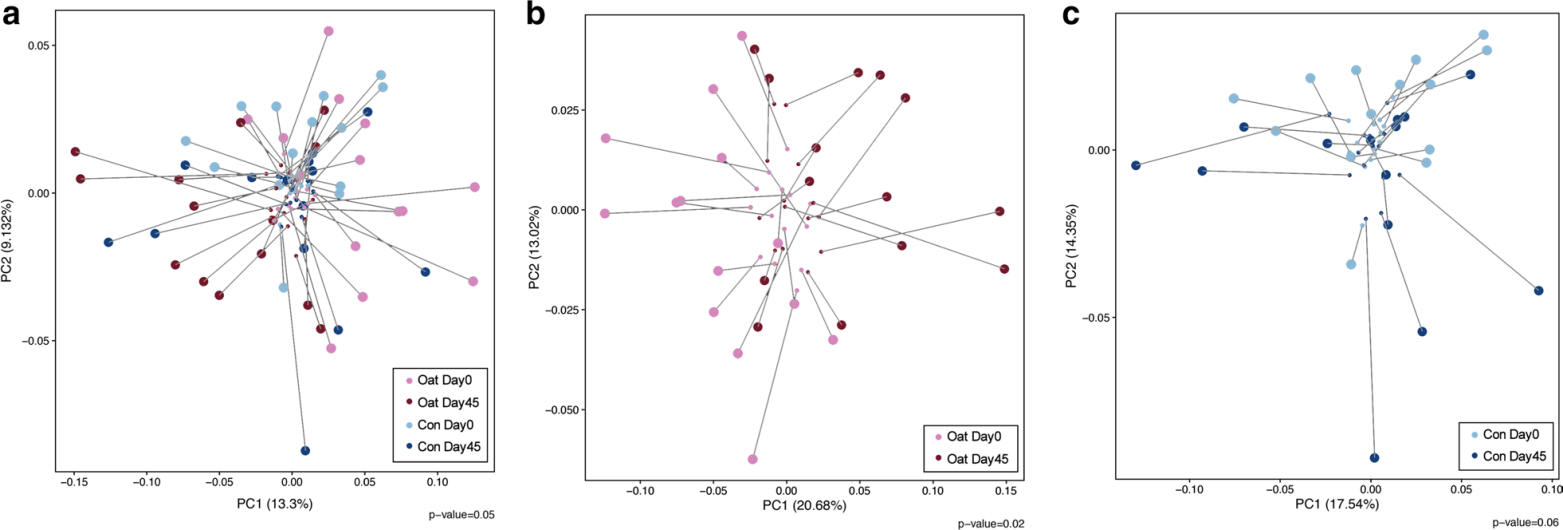
The concordance between the structural alteration of 448 OTUs and the alleviation of hypercholesterolemia phenotypes. Procrustes analysis combined the principal coordinate analysis from 448 OTUs (end of lines with big solid symbols) with the principal coordinate analysis from the lipid variables (end of lines with small solid symbols) for (**a**) all participants, (**b**) oatmeal group, (**c**) control group. *P* value was from 999 Monte-Carlo simulations. PC1, principal coordinate 1; PC2, principal coordinate 2. Oat Day 0: baseline in oatmeal group; Oat Day 45: endpoint in oatmeal group; Con Day 0: baseline in control group; Con Day 45: endpoint in control group

**Fig. 5:**
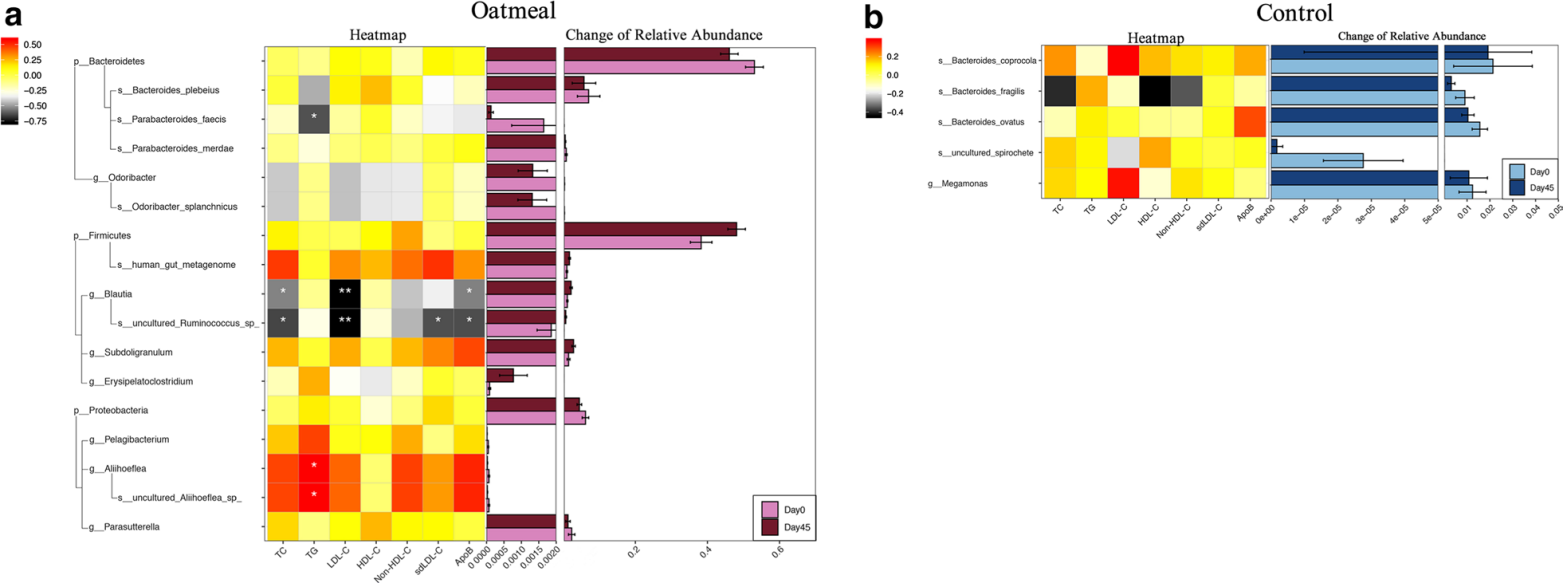
Identification of the major microbiota associated with the improvements of clinical phenotypes in the (**a**) oatmeal and (**b**) control group. Heatmaps showed the results of spearman correlations analysis. Only microbiota that were significantly changed after interventions (according to the result of LEfSe) were shown. The color represented positive (black) or negative (red) correlations with the improvements of lipid profiles, respectively, and the *P* value was denoted as follows: *P < 0.05, **P < 0.01

We then made a further inquiry in the association between the above significantly altered microbiota and the changes of lipid profiles following interventions. The Procrustes analysis demonstrated a trend (*P* = 0.05) of association between microbiota composition alteration and lipid profiles changes in all participants during study period (Fig. 4a). And respective Procrustes analysis in two groups further supported the concordance of compositional alteration of microorganism and the alleviation of hypercholesterolemia phenotypes after oatmeal consumption (Fig. 4b, c). Next, on the basis of significantly changed microbiota found respectively in two groups, Spearman’s correlation analysis was conducted at phylum, genus and species levels to identify if there were any specific intervention-induced microbiota alterations that would be potentially responsible for the improvements of lipid profiles (Fig. 5). The results turned out that, in the oatmeal group, Blautia genus stood out, with its enrichment being positively associated with the improvements of TC, LDL-C and apoB after oatmeal consumption. Besides, the decreases of Parabacteroides faecis species and Aliihoeflea genus following oatmeal consumption were observed to be positively and negatively related with the improvement of TG, respectively. Whereas, in the control group, the results showed that no significant association was seen between the microbiota alterations and changes of lipid.

## Discussion

Our study demonstrated that there was no significant difference in the compositional microbiota alteration of overall α-diversity or β-diversity between oatmeal and control group throughout study, nor there be any significant difference in the relative abundance alteration at different taxonomies between two groups. However, the within-group comparisons suggested a potentially critical role of Firmicutes phylum in the oatmeal-induced characteristic of microbiota alteration as a core positive responder. Moreover, the further within-group results of correlation analysis hinted that, the enrichment of Blautia genus was worth of being researched as an underlying mechanism of oatmeal-induced cholesterol lowering, since it showed a significantly tight relationship with the reduction of TC, LDL-C and apoB in the oatmeal group.

Similar to a previous clinical oatmeal feeding study [11], our data showed no significant increase in the overall diversity of microbiota community after oatmeal consumption, which challenged the previous conception that greater diversity implies better health [21]. However, these negative results of the microbiota alteration of overall α or β-diversity, to some extent, could be an increment evidence supporting the emerging notion that a mild alteration in microbial composition might be sufficient to drive significant functional changes [22, 23]. Besides, different from the previous reports [5, 7, 12], we did not observed any prebiotic effect of oatmeal in this study.

A transformation of a Bacteroidetes-predominated pattern to a Firmicutes-predominated pattern was seen after oatmeal consumption. And the result of within-group LEfSe further supported the important role of Firmicutes phylum in the oatmeal-induced microbiota characteristics. Both Firmicutes and Bacteroidetes were the predominant bacterial phyla in the large intestine of healthy human which were able to ferment dietary fiber [24]. Bacteroidetes phylum possessed tremendous numbers of carbohydrate-active enzymes (CAZymes) and was found elaborated a few thousand enzyme combinations to break down glycans [24]. However, despite a far less abundance of CAZymes than Bacteroides, Firmicutes phylum was found being actively respond to changes of major dietary carbohydrate in various clinical trials, which showed a consistency with our study [25, 26]. One of a current point of view was that Firmicutes phylum played a role in breaking down specific kinds of carbohydrate like fermentable fiber (“as specialists for a select set of glycans catabolism”) under a context of Bacteroides phylum being the primary polysaccharides degraders (“generalists for glycan catabolism”) [24]. Thus, the “nutritionally highly specialized” characteristic of Firmicutes phylum due to its ability of producing specific kinds of CAZymes for oatmeal degrading, might be a reason explaining the enrichment of Firmicute after oatmeal consumption in our study.

Further Spearman’s correlation analysis suggested that the enrichment of Blautia genus responding to oatmeal consumption might underlie the oatmeal-induced improvements of lipid profiles. Blautia genus was a short-chain fatty acid (SCFA) producing genus, and it was reported to be a beneficial bacterium negatively associated with metabolic syndromes, with its mainly biological function thought to be producing butyrate [27]. Although we did not test the concentration of SCFA in fecal or serum samples, the observed enrichment of Blautia genus following oatmeal consumption matched up to the result of increased fecal butyrate concentration from previously reported in vitro fermentation studies and animal experiments [28–30]. Besides, considerable evidences have reported that SCFA, especially butyrate, might participate in cholesterol metabolism in various ways, such as inhibiting intestinal cholesterol absorption [31], increasing fecal excretion of bile acid [32, 33] as well as promoting uptake and metabolize cholesterol from blood [32]. Consequently, together with some similar evidences, which also showed a positive relationship between enrichment of Blautia genus and improved lipid profiles [34], we speculated that the enrichment of Blautia genus might play its significant role in improving lipid profiles by inducing a subsequently elevated production of butyrate. In addition, a majority of bacterial phylotypes, which associated tightly with a high fecal coprostanol level, were found basically being from the Lachnospiraceae and Ruminococcaceae family, suggesting a potentially coprostanoligenic activity of members from these families [35]. Therefore, direct participating in the process of converting cholesterol to coprostanol may also one of the underlying mechanisms of Blautia genus enrichment toward cholesterol lowering.

Besides, we also found the oatmeal-induced decrease of two genus (Parabacteroides and Aliihoeflea) shared opposite correlations with the decrease of TG. This opposite microbiota-regulating effect toward TG following oatmeal consumption seemingly provided a great explanation for the currently recognized notion that oatmeal was ineffective in regulating serum TG. And it again, together with the results of Procrustes analysis, supported our hypothesis that microbiota-regulating effect might be an underlying mechanism of oatmeal induced improvements of lipid profiles.

However, there was no denying that the small sample size has limited our ability in detecting the between-group differences in the alterations of microbiota composition, and results from the within-group comparisons could only provide underlying clues involving. This was one of the drawbacks of our study, and it failed us to draw exactly reliable conclusions in the independent microbiota-manipulating effect of oatmeal consumption. Moreover, higher concentrations of sdLDL-C and TG in the oatmeal group at baseline might also cause some confounding effects in the oatmeal-induced microbiota-regulating effect. Because, if the hypercholesterolemia phenotype did associate with the gut microbiota composition as we expected, a more severe hypercholesterolemia status might lead to a more anomalous microbiome composition from that of healthy population, and thus, left more “space” for improvements. In addition, due to limitations in quality and quantity of fecal and serum samples as well as lacking of collecting urinary samples, we did not detect the concentrations of SCFA or any other microbiota metabolites in these samples, nor did we separate any significantly altered specific strains from oatmeal group for further corresponding verificational animal experiments, which might be stronger evidences in supporting our arguments.

## Conclusions

Eating 80 g/day oatmeal for 45 days did not induce notable alterations in the overall microbiota structure, yet a positive responding of Firmicute phylum might be a critical characteristic of its microbiota alteration. And on the basis of the tight association between the oatmeal-induced enrichment of Blautia genus and the lowering of TC, LDL-C as well as apoB, microbiota-regulating effect might be a promising underlying mechanism of oatmeal-induced cholesterol lowering. However, with drawbacks of small sample size as well as lacking of evidences from further verificational experiments, current results should be taken cautiously.

## Supplementary information

**Additional file 1.: Supplementary Table 1.** Results of post-hoc power calculations. **Supplementary Table 2.** Changes of physical activities in the oatmeal and control group throughout the study. **Supplementary Table 3.** Alterations of microbiota composition (relative abundance [%]) at phylum level.

## Data Availability

The raw reads of 16SrRNA were deposited into the NCBI Sequence Read Archive (SRA) database (Accession number: SRP230372). Other datasets used and/or analyzed during the current study are available from the corresponding author on reasonable request.

## Abbreviations

TC: Total cholesterol
TG: Triglycerides
BMI: Body mass index
LDL-C: Low-density lipoprotein cholesterol
HDL-C: High-density lipoprotein cholesterol
sdLDL-C: Small dense low-density lipoprotein cholesterol
apoB: Apolipoprotein B
non-HDL-C: Non-high-density lipoprotein cholesterol
DNA: Deoxyribonucleic acid
PCR: Polymerase chain reaction
OTU: Operational taxonomic units
PCoA: Principal coordinate analysis
UPGMA: Unweighted pair-group method with arithmetic mean
FDR: False discovery rate
SE: Standard error
ANCOVA: Analysis of covariance
LEfSe: Linear discriminant analysis effect size
LDA: Linear-discriminant analysis
CAZymes: Carbohydrate-active enzymes
SCFA: Short-chain fatty acid

## References

[1] Koropatkin NM, Cameron EA, Martens EC (2012). How glycan metabolism shapes the human gut microbiota. Nat Rev Microbiol.

[2] Long SL, Gahan CGM, Joyce SA (2017). Interactions between gut bacteria and bile in health and disease. Mol Asp Med.

[3] Gerard P, Lepercq P, Leclerc M, Gavini F, Raibaud P, Juste C (2007). Bacteroides sp. strain D8, the first cholesterol-reducing bacterium isolated from human feces. Appl Environ Microbiol.

[4] Noh DO, Kim SH, Gilliland SE (1997). Incorporation of cholesterol into the cellular membrane of Lactobacillus acidophilus ATCC 43121. J Dairy Sci.

[5] Shen RL, Dang XY, Dong JL, Hu XZ (2012). Effects of oat beta-glucan and barley beta-glucan on fecal characteristics, intestinal microflora, and intestinal bacterial metabolites in rats. J Agric Food Chem.

[6] Wilczak J, Blaszczyk K, Kamola D, Gajewska M, Harasym JP, Jalosinska M (2015). The effect of low or high molecular weight oat beta-glucans on the inflammatory and oxidative stress status in the colon of rats with LPS-induced enteritis. Food Funct.

[7] Connolly ML, Tuohy KM, Lovegrove JA (2012). Wholegrain oat-based cereals have prebiotic potential and low glycaemic index. Br J Nutr.

[8] Connolly ML, Lovegrove JA, Tuohy KM (2010). In vitro evaluation of the microbiota modulation abilities of different sized whole oat grain flakes. Anaerobe.

[9] Carlson JL, Erickson JM, Hess JM, Gould TJ, Slavin JL (2017). Prebiotic dietary fiber and gut health: comparing the in vitro fermentations of beta-glucan, inulin and xylooligosaccharide. Nutrients.

[10] Hughes SA, Shewry PR, Gibson GR, McCleary BV, Rastall RA (2008). In vitro fermentation of oat and barley derived beta-glucans by human faecal microbiota. FEMS Microbiol Ecol.

[11] Li J, Hou Q, Zhang J, Xu H, Sun Z, Menghe B (2017). Carbohydrate staple food modulates gut microbiota of mongolians in China. Front Microbiol.

[12] Connolly ML, Tzounis X, Tuohy KM, Lovegrove JA (2016). Hypocholesterolemic and prebiotic effects of a whole-grain oat-based granola breakfast cereal in a cardio-metabolic “at risk” population. Front Microbiol.

[13] Li J, Xu H, Sun Z, Hou Q, Kwok L-Y, Laga W (2016). Effect of dietary interventions on the intestinal microbiota of Mongolian hosts. Sci Bull.

[14] Ye MY, Sun JQ, Chen YQ, Ren Q, Zhao YF, Pan YR (2020). Response of serum LDL cholesterol to oatmeal consumption depends on CYP7A1_rs3808607 genotype in Chinese. Asia Pac J Clin Nutr.

[15] Yang YX, Wang GY, Pan XC (2002). China food composition 2002.

[16] Huang W, Jiang X (2016). Profiling of sediment microbial community in dongting lake before and after impoundment of the three gorges dam. Int J Environ Res Public Health.

[17] Bolger AM, Lohse M, Usadel B (2014). Trimmomatic: a flexible trimmer for Illumina sequence data. Bioinformatics.

[18] Magoc T, Salzberg SL (2011). FLASH: fast length adjustment of short reads to improve genome assemblies. Bioinformatics.

[19] Edgar RC, Haas BJ, Clemente JC, Quince C, Knight R (2011). UCHIME improves sensitivity and speed of chimera detection. Bioinformatics.

[20] Price MN, Dehal PS, Arkin AP (2010). FastTree 2–approximately maximum-likelihood trees for large alignments. PLoS ONE.

[21] Le Chatelier E, Nielsen T, Qin J, Prifti E, Hildebrand F, Falony G (2013). Richness of human gut microbiome correlates with metabolic markers. Nature.

[22] Liu Y, Wang Y, Ni Y, Cheung CKY, Lam KSL, Wang Y (2020). Gut microbiome fermentation determines the efficacy of exercise for diabetes prevention. Cell Metab.

[23] Bercik P, Denou E, Collins J, Jackson W, Lu J, Jury J (2011). The intestinal microbiota affect central levels of brain-derived neurotropic factor and behavior in mice. Gastroenterology.

[24] Sheridan PO, Martin JC, Lawley TD, Browne HP, Harris HMB, Bernalier-Donadille A (2016). Polysaccharide utilization loci and nutritional specialization in a dominant group of butyrate-producing human colonic Firmicutes. Microb Genom.

[25] Claesson MJ, Jeffery IB, Conde S, Power SE, O'Connor EM, Cusack S (2012). Gut microbiota composition correlates with diet and health in the elderly. Nature.

[26] Wu GD, Chen J, Hoffmann C, Bittinger K, Chen YY, Keilbaugh SA (2011). Linking long-term dietary patterns with gut microbial enterotypes. Science.

[27] Berni Canani R, Sangwan N, Stefka AT, Nocerino R, Paparo L, Aitoro R (2016). Lactobacillus rhamnosus GG-supplemented formula expands butyrate-producing bacterial strains in food allergic infants. ISME J.

[28] Tsitko I, Wiik-Miettinen F, Mattila O, Rosa-Sibakov N, Seppanen-Laakso T, Maukonen J (2019). A small in vitro fermentation model for screening the gut microbiota effects of different fiber preparations. Int J Mol Sci.

[29] van Zanten GC, Knudsen A, Roytio H, Forssten S, Lawther M, Blennow A (2012). The effect of selected synbiotics on microbial composition and short-chain fatty acid production in a model system of the human colon. PLoS ONE.

[30] Drzikova B, Dongowski G, Gebhardt E (2005). Dietary fibre-rich oat-based products affect serum lipids, microbiota, formation of short-chain fatty acids and steroids in rats. Br J Nutr.

[31] Chen Y, Xu C, Huang R, Song J, Li D, Xia M (2018). Butyrate from pectin fermentation inhibits intestinal cholesterol absorption and attenuates atherosclerosis in apolipoprotein E-deficient mice. J Nutr Biochem.

[32] Zhao Y, Liu J, Hao W, Zhu H, Liang N, He Z (2017). Structure-specific effects of short-chain fatty acids on plasma cholesterol concentration in male syrian hamsters. J Agric Food Chem.

[33] Fushimi T, Suruga K, Oshima Y, Fukiharu M, Tsukamoto Y, Goda T (2006). Dietary acetic acid reduces serum cholesterol and triacylglycerols in rats fed a cholesterol-rich diet. Br J Nutr.

[34] Ren SM, Mei L, Huang H, Cao SF, Zhao RH, Zheng PY (2019). Correlation analysis of gut microbiota and biochemical indexes in patients with non-alcoholic fatty liver disease. Chin J Hepatol.

[35] Antharam VC, McEwen DC, Garrett TJ, Dossey AT, Li EC, Kozlov AN (2016). An integrated metabolomic and microbiome analysis identified specific gut microbiota associated with fecal cholesterol and coprostanol in clostridium difficile infection. PLoS ONE.

