# Supplementary material for: Oatmeal induced gut microbiota alteration and its relationship with improved lipid profiles: a secondary analysis of a randomized clinical trial
Source: Nutr Metab (Lond). 2020 Oct 8;17:85. doi: 10.1186/s12986-020-00505-4 (PMC7542720; doi:10.1186/s12986-020-00505-4)
Supplement: Supplementary file 1 — Additional file 1.: Supplementary Table 1. Results of post-hoc power calculations. Supplementary Table 2. Changes of physical activities in the oatmeal and control group throughout the study. Supplementary Table 3. Alterations of microbiota composition (relative abundance [%]) at phylum level. [file 12986_2020_505_MOESM1_ESM.docx]

**Supplementary Table**

**(Line 457) Supplementary Table 1. Results of post-hoc power calculations.**

| Variable | Effect Size | Standard Deviation | Power |
| --- | --- | --- | --- |
|  |  |  |  |
| Shannon Index^†^ | 0.166 | 0.091 | 0.996 |
| Ace Index^†^ | 19.408 | 7.581 | 1.000 |
| Whole Community^‡^ | | | |
| Within Oatmeal Group | - | - | 0.001 |
| Within Control Group | - | - | 0.001 |
| Between-group Comparison at Endpoint | - | - | 0.006 |

†. The pwr package in R (3.6.0) was used to conduct post-hoc power calculations of Shannon index and Ace index based on the between-group difference of alterations by using independent *t* test (two-tailed), with the significance level set to 0.05 and a sample size of 14 per group. ‡. The HMP package in R (3.6.0) was used to conduct post-hoc power calculations under Dirichlet-Multinomial model, with the significance level set to 0.05, a sample size of 14 per group and an average read of 30000 per sample.

**(Line 526) Supplementary Table 2. Changes of physical activities in the oatmeal and control group throughout the study.**

| Variables | Group | Day 0 | Day 45 | *P*  Value | Change from baseline | *P* Value time by treated |
| --- | --- | --- | --- | --- | --- | --- |
| Every-week working day  (d/w) | Oatmeal | 5.18±0.10 | 5.18±0.10 | 0.22^‡^ | 0.00±0.09 | 0.60^†^ |
|  | Control | 3.86±0.58 | 3.96±0.58 | 0.68^‡^ | 0.11±0.17 |  |
| Every-day working time  (h/d) | Oatmeal | 8.86±0.41 | 8.79±0.33 | 0.23^‡^ | -0.07±0.24 | 0.19^†^ |
|  | Control | 6.14±0.94 | 6.43±0.96 | 0.43^‡^ | 0.29±0.16 |  |
| Sitting time  (h/d） | Oatmeal | 7.54±0.73 | 7.36±0.61 | 0.59 | -0.18±0.52 | 1.00 |
|  | Control | 6.46±0.64 | 6.29±0.57 | 0.06 | -0.18±0.33 |  |
| Commuting time  (min/d） | Oatmeal | 74.6±10.5 | 58.6±10.1 | 0.01^‡^ | -16.1±7.5 | 0.87^†^ |
|  | Control | 91.4±17.4 | 75.0±14.5 | 0.08^‡^ | -16.4±8.7 |  |
| Weekly frequency of medium intensity exercise | Oatmeal | 0.17±0.10 | 0.12±0.07 | 0.02^‡^ | -0.05±0.08 | 0.73^†^ |
|  | Control | 0.24±0.08 | 0.19±0.08 | 0.09^‡^ | -0.05±0.08 |  |
| Weekly frequency of heavy intensity exercise | Oatmeal | 0.06±0.03 | 0.04±0.03 | 0.29^‡^ | -0.02±0.03 | 0.23^†^ |
|  | Control | 0.11±0.05 | 0.00±0.00 | 0.02^‡^ | -0.11±0.05 |  |
| House working time  (min/d) | Oatmeal | 36.9±13.6 | 27.5±9.0 | 0.06^‡^ | -9.43±6.41 | 0.91^†^ |
|  | Control | 66.8±17.0 | 70.0±19.2 | 0.69^‡^ | 3.21±14.14 |  |
| Sleeping time  (h/d) | Oatmeal | 6.64±0.18 | 6.68±0.12 | 0.89^‡^ | 0.04±0.14 | 0.33^†^ |
|  | Control | 6.79±0.25 | 6.61±0.27 | 0.61^‡^ | -0.18±0.10 |  |

Values were presented as mean ± SE.†. *P* value time by treated was obtained from Mann–Whitney U test (two-tailed), otherwise, it was obtained from independent-sample *t* test (two-tailed). ‡. *P* value was obtained from Wilcoxon signed rank test (two-tailed), otherwise, it was obtained from paired *t* test (two-tailed).

**(Line 605) Supplementary Table 3. Alterations of microbiota composition (relative abundance [%]) at phylum level.**

| Phylum | Oatmeal | | |  | Control | | | *P* Value  Time by Treated |
| --- | --- | --- | --- | --- | --- | --- | --- | --- |
|  | Day 0 | Day 45 | *P* Value |  | Day 0 | Day 45 | *P* Value |  |
| Above 1 % of community | | | | | | | | |
| Bacteroidetes | 53.04±2.46 | 46.09±2.42 | 0.02 |  | 55.10±1.64 | 49.63±2.05 | 0.07 | 0.70 |
| Firmicutes | 38.26±2.99 | 48.10±2.44 | <0.01 |  | 39.13±1.91 | 42.22±3.36 | 0.50 | 0.23 |
| Proteobacteria | 6.17±0.90 | 4.46±0.64 | 0.03^‡^ |  | 4.67±0.49 | 4.63±0.50 | 0.25^‡^ | 0.43^†^ |
| Fusobacteria | 1.46±1.41 | 0.04±0.03 | 0.55^‡^ |  | 0.19±0.15 | 2.25±1.37 | 0.16^‡^ | 0.31^†^ |
| Between 0.1% and 1% of community | | | | | | | | |
| Actinobacteria | 0.72±0.36 | 0.82±0.41 | 0.60^‡^ |  | 0.53±0.19 | 0.74±0.22 | 0.08^‡^ | 0.35^†^ |
| Verrucomicrobia | 0.12±0.07 | 0.06±0.03 | 0.53^‡^ |  | 0.24±0.17 | 0.48±0.33 | 0.40^‡^ | 0.43^†^ |
| Tenericutes | 0.16±0.13 | 0.20±0.15 | 0.27^‡^ |  | 0.12±0.08 | 0.01±0.01 | 0.18^‡^ | 0.25^†^ |
| Less than 0.1% of community | |  |  |  |  |  |  |  |
| Cyanobacteria | 0.08±0.048 | 0.20±0.20 | 0.32^‡^ |  | - | - | 1.00^‡^ | 0.77^†^ |
| Synergistetes | 0.002±0.002 | 0.010±0.005 | 0.21^‡^ |  | 0.011±0.007 | 0.026±0.013 | 0.23^‡^ | 0.77^†^ |
| Lentisphaerae | 0.014±0.008 | 0.010±0.005 | 0.59^‡^ |  | 0.004±0.004 | 0.001±0.001 | 0.32^‡^ | 0.57^†^ |
| Saccharibacteria | 0.006±0.003 | - | 0.45^‡^ |  | 0.007±0.004 | 0.010±0.003 | 0.39^‡^ | 0.70^†^ |

Values were presented as mean±SE. †. *P* value time by treated was obtained from Mann–Whitney U test (two-tailed), otherwise, it was obtained from independent-sample *t* test (two-tailed). ‡. *P* value was obtained from Wilcoxon signed rank test (two-tailed), otherwise, it was obtained from paired *t* test (two-tailed).
